# Case Report: Catamenial pneumothorax: clinical characteristics, diagnosis, and management — a report of two cases with a review of the literature

**DOI:** 10.3389/fmed.2026.1984821

**Published:** 2026-10-01

**Authors:** Wen-Song Shi, Yueyao Liu, He Liu, Yu-Zhui Hu, Huijuan Fang, Meng-Hao Zhou, Liang-Yi Dong, Hui-Yu Zheng, Hongjuan Li

**Affiliations:** 1 Department of Thoracic Surgery, The Fifth Clinical Medical College of Henan University of Chinese Medicine, Zhengzhou People’s Hospital, Zhengzhou, Henan, China; 2 The Fifth Clinical Medical College of Henan University of Chinese Medicine, Zhengzhou, Henan, China; 3 Department of Gynecology, The Fifth Clinical Medical College of Henan University of Chinese Medicine, Zhengzhou People’s Hospital, Zhengzhou, Henan, China; 4 Department of Geriatrics, Zhengzhou Ninth People’s Hospital, Zhengzhou, Henan, China; 5 Department of Pathology, The Fifth Clinical Medical College of Henan University of Chinese Medicine, Zhengzhou People’s Hospital, Zhengzhou, Henan, China

**Keywords:** catamenial pneumothorax, endometriosis, pneumothorax, recurrent pneumothorax, reproductive age

## Abstract

Pneumothorax is a pathological condition in which gas enters the pleural cavity due to trauma, surgery, positive-pressure ventilation, thoracentesis, or other causes, leading to compression and collapse of the lung tissue. It exhibits a male predominance and can occur in all age groups. Catamenial pneumothorax, also referred to as thoracic endometriosis syndrome-associated pneumothorax, is a rare subtype. It is characterized by spontaneous and recurrent pneumothorax occurring in women of reproductive age during the perimenstrual period. Clinicians often fail to associate pneumothorax with the menstrual cycle, resulting in a high rate of misdiagnosis in clinical practice, which in turn leads to recurrent episodes and protracted clinical courses. This article presents two case reports, summarizes and analyzes their clinical features, and explores diagnostic and therapeutic strategies, with the aim of enhancing awareness of the association between recurrent pneumothorax and menstruation in women of reproductive age, thereby reducing underdiagnosis and misdiagnosis.

## 1 Introduction

Catamenial pneumothorax, also termed thoracic endometriosis – related pneumothorax (1, 2), is a rare and distinctive subtype of spontaneous pneumothorax that occurs exclusively in women of reproductive age. Its defining clinical feature is a clear temporal relationship between the onset of pneumothorax and the menstrual cycle. This condition was first reported by Maurer et al. (3) in 1958 and has since garnered increasing attention within the medical community. In 1972, the term “catamenial pneumothorax” was formally proposed by Lillington et al. (4). Currently, the predominant pathological mechanism is widely considered to be intrathoracic endometrial implantation (1, 5, 6). According to the literature, catamenial pneumothorax accounts for approximately 3%–6% of all spontaneous pneumothoraces in women, with right-sided involvement occurring in 85%–95% of cases. The onset of symptoms most frequently occurs within the interval from 24–72 hours before menstruation through 72 hours after its onset. Nevertheless, some investigators have suggested that the true incidence of this entity may be substantially underestimated, owing to its low rate of clinical recognition.

At present, the principal clinical challenge posed by catamenial pneumothorax lies in its high misdiagnosis rate and delayed diagnosis, which stem largely from insufficient awareness of this entity among clinicians. When encountering women of reproductive age with pneumothorax, most primary care physicians fail to actively establish a temporal association between the onset of pneumothorax and the menstrual cycle, whereas patients themselves rarely volunteer such a correlation. Previous studies (7, 8) have demonstrated that the interval from initial symptom onset to definitive diagnosis is frequently prolonged from several months to even years, during which patients often undergo repeated non-curative interventions and experience exceedingly high rates of recurrence. It should be emphasized that if simple tube thoracostomy is performed without addressing the underlying etiology, if conventional video-assisted thoracoscopic surgery (VATS) is undertaken but diaphragmatic lesions or defects are inadvertently overlooked, or if postoperative endometriosis-targeted medical therapy is not administered, the postoperative recurrence rate remains considerably elevated, thereby significantly compromising patients’ long-term quality of life (9, 10).

This study presents a retrospective analysis of two pathologically confirmed cases of catamenial pneumothorax, with a systematic review of their clinical characteristics, radiological findings, intraoperative observations, and histopathological features. In conjunction with the relevant literature, the pathogenesis and multidisciplinary team (MDT) – based therapeutic strategies are further explored. The primary objective of this report is to reinforce clinical awareness among practicing physicians that a detailed menstrual history must be routinely elicited in women of reproductive age presenting with recurrent pneumothorax, so as to minimize the risk of missed diagnosis and misdiagnosis and ultimately improve the overall prognosis of affected patients.

## 2 Cases

### 2.1 Case 1

A 37-year-old unmarried nulliparous female patient presented to the emergency department of our hospital on April 22, 2025, with a chief complaint of right-sided chest pain lasting for over half an hour. Approximately 30 minutes before presentation, the patient experienced sudden onset of persistent pain in the right chest, which was exacerbated by deep breathing, accompanied by chest tightness and dyspnea that worsened with physical activity. No fever, cough, or other accompanying symptoms were reported. For further diagnostic clarification, a chest CT scan was promptly performed in the emergency setting, which revealed a small right–sided pneumothorax (Figure 1A) and a ground–glass nodule measuring approximately 6 × 5 mm in the posterior segment of the right upper lobe, with a mean CT attenuation value of approximately −673 HU. Based on these findings, a preliminary diagnosis of spontaneous pneumothorax and solitary pulmonary nodule was established, and the patient was subsequently admitted to our department for further management.

**Figure 1:**
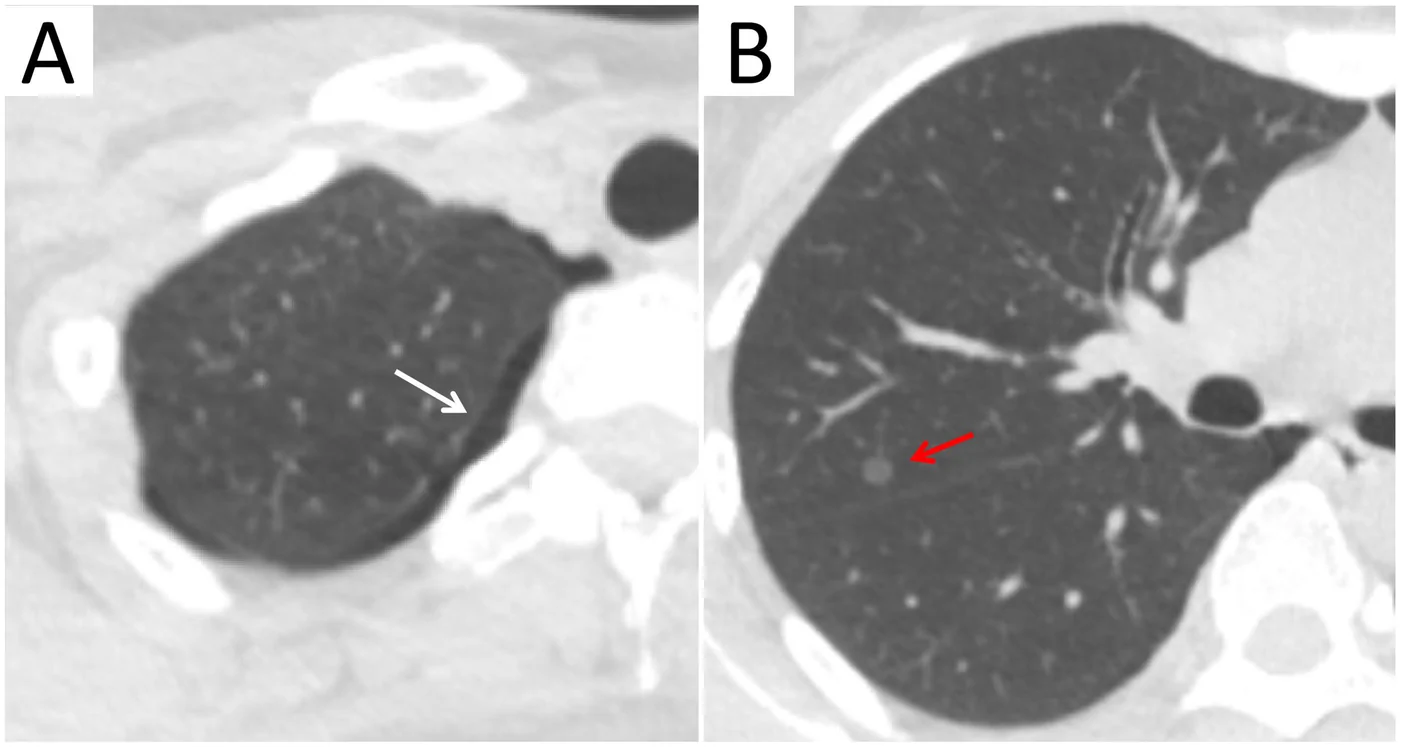
Radiological findings of case 1. **(A)** The white arrow indicates the margin of pulmonary compression. **(B)** The red arrow denotes the ground-glass nodule.

The patient had undergone left thyroid lobectomy and isthmectomy for a “thyroid nodule” four years prior to the current admission. Postoperative histopathological examination revealed papillary thyroid carcinoma, for which the patient had been receiving regular oral levothyroxine replacement therapy since surgery. Of note, the patient had experienced recurrent episodes of similar chest pain since 2021, with an average frequency of two to three episodes per year, which had not been taken seriously and had not prompted further diagnostic evaluation at that time.

After admission, because the pneumothorax was estimated at approximately 10%, conservative management consisting of oxygen supplementation and infection prophylaxis was initiated. Relevant biochemical tests showed no significant abnormalities. Later, after review and comparison with previous records/imaging, a persistent ground-glass nodule in the right upper lobe was identified (Figure 1B), and an early-stage neoplastic nodule was considered highly likely. Therefore, on April 26, 2025, the patient underwent uniportal video-assisted thoracoscopic pulmonary wedge resection. Exploration revealed a small number of pulmonary bullae at the lung apex, which were wedge-resected; an intraoperative water-seal test showed no obvious air leakage. The ground-glass nodule was localized preoperatively by CT-guided hookwire placement and was also wedge-resected. Postoperative pathology revealed pulmonary bullae at the lung apex and adenocarcinoma *in situ* (AIS) of the lung in the ground-glass nodule. Follow-up frontal chest radiography on April 30, 2025 showed satisfactory lung re-expansion. The chest tube was successfully removed on postoperative day 4, and the patient was discharged.

At the first postoperative month follow-up, chest imaging revealed satisfactory lung re-expansion. However, during the second postoperative month (July 13, 2025), the patient presented again with chest pain. Emergency chest CT demonstrated a right-sided hydropneumothorax, postoperative changes in the right upper lobe, patchy increased opacity in the right middle lobe, a small left-sided pleural effusion, and nodular calcific foci in the right breast. An urgent tube thoracostomy was performed; after five days of drainage, follow-up chest radiography confirmed satisfactory lung re-expansion, and the patient was discharged with the chest tube removed. Approximately three months postoperatively, the patient re-presented with chest pain at a local hospital, where chest CT revealed a right-sided pneumothorax occupying approximately 30% of the hemithorax. Upon admission, the patient strongly requested conservative management, and oxygen supplementation with prophylactic anti-infective therapy was administered.

In view of the recurrent pneumothoraces, a thorough literature review raised the suspicion of catamenial pneumothorax. A gynecological consultation was subsequently obtained, which revealed the following: the patient reported regular menstrual cycles of 26–28 days, with a menstrual flow duration of six days, accompanied by pronounced dysmenorrhea and lumbosacral soreness; while bowel and bladder functions remained normal. A chronologic review of her menstrual history and pneumothorax episodes demonstrated the following temporal associations: menstruation on July 29, 2024, with right-sided pneumothorax onset on July 31, 2024; menstruation on April 20, 2025, with right-sided pneumothorax onset on April 22, 2025 (followed by video-assisted thoracoscopic wedge resection and bullectomy at our institution on April 26, 2025); menstruation on June 15, 2025, with right-sided pneumothorax diagnosed on June 19, 2025; and menstruation on July 11, 2025, with recurrence of right-sided pneumothorax on July 12, 2025.

Pelvic ultrasonography for deep infiltrating endometriosis revealed multiple cystic echoes in the bilateral adnexal regions, the largest measuring approximately 49 × 42 mm, with extremely poor internal echogenicity, a positive “kissing” sign, and absence of sliding sign between the cysts and the posterior uterine wall. Pelvic effusion and multiple cystic lesions in the bilateral adnexal regions (suspected chocolate cysts) with localized adhesions were also noted. A clinical diagnosis of bilateral ovarian endometriotic cysts was tentatively established. The patient opted for conservative management and was treated with subcutaneous leuprorelin acetate injections administered every four weeks for a planned duration of three to six months. Concurrently, immunohistochemical staining for estrogen receptor (ER) and progesterone receptor (PR) was performed on the previously resected pulmonary specimens.

Subsequently, communication with the Department of Pathology was initiated to perform additional immunohistochemical staining for ER and PR on the resected apical lung specimen. The results revealed the following: ER exhibited weak-to-moderate positivity in approximately 20% of stromal cells, while PR demonstrated moderate positivity in approximately 40% of stromal cells. Following consultation with the gynecological service, a clinical diagnosis of catamenial pneumothorax was established. Accordingly, regular administration of leuprorelin acetate was initiated. One month after the initiation of therapy, the patient experienced another menstrual episode, and a chest radiograph revealed a small right-sided pneumothorax, which was managed conservatively. Subsequently, with continuous regular use of leuprorelin over a six-month period (One injection every 28 days, one vial per dose), no menstrual bleeding occurred, and serial follow-up imaging demonstrated no recurrent pneumothorax during this interval. However, the patient developed significant menopausal-type symptoms, including hot flushes, excessive diaphoresis, and insomnia, leading to self-discontinuation of the medication. Follow-up information indicated that in July 2026, upon resumption of menstruation, the patient again experienced right-sided chest pain, and a local hospital examination confirmed a small right-sided pneumothorax, which was again managed conservatively. The diagnostic and therapeutic timeline for Case 1 is summarized in Figure 2.

**Figure 2:**
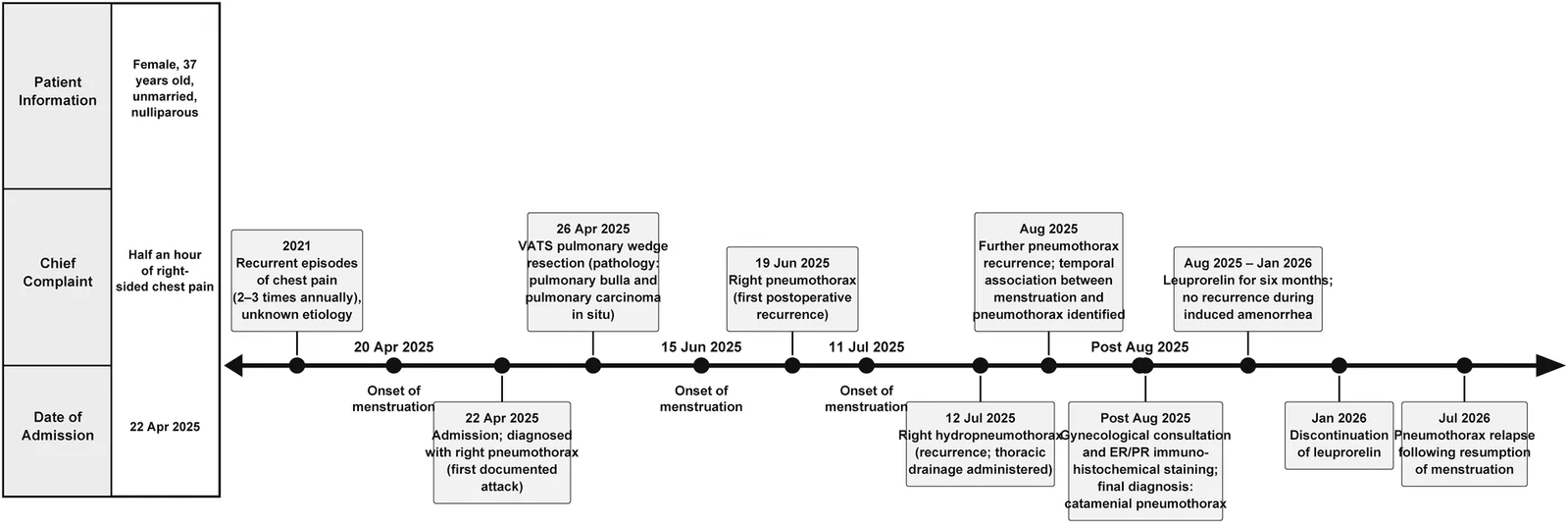
Timeline of the clinical course of case 1.

### 2.2 Case 2

A 41-year-old married female patient, mother of two daughters, was admitted to our hospital on June 27, 2026, with a chief complaint of right-sided chest pain of three days’ duration. Approximately three days before admission, the patient developed right-sided chest pain without identifiable precipitating factors, accompanied by chest tightness and dyspnea, which were aggravated by physical activity. The symptoms were initially disregarded and showed no significant resolution over time. The patient subsequently presented to an outside hospital, where a right-sided pneumothorax was identified, and she was then referred to our institution. Upon outpatient evaluation, the patient was admitted with a diagnosis of spontaneous pneumothorax. Since the onset of symptoms, the patient had remained alert with stable general condition, normal bowel and bladder functions, and no appreciable weight loss.

The patient had undergone surgical intervention for a “thyroid nodule” at an outside hospital ten years prior to the current admission. Postoperative histopathological examination revealed thyroid carcinoma, for which she had been receiving regular oral levothyroxine replacement therapy since surgery. Nine years ago, the patient had a history of right-sided pleuritis and pleural effusion, which improved following anti-infective therapy, although the specific details of the illness remained unclear. Six years ago, she underwent surgical treatment for an “ovarian cyst” at another institution. The patient also had a three-year history of recurrent right-sided pneumothorax managed conservatively, without further diagnostic investigation. One month prior to the current admission (May 18, 2026), she was hospitalized with a chief complaint of right-sided chest pain lasting for over one week. Chest CT at that time revealed a spontaneous right-sided moderate-sized pneumothorax with approximately 60% local lung compression, accompanied by a small right-sided pleural effusion. Tube thoracostomy was performed. In view of the patient's history of recurrent pneumothorax and its temporal association with the menstrual cycle, a clinical diagnosis of catamenial pneumothorax was established. Following gynecological consultation, pelvic ultrasonography was further performed, which revealed a cystic echo measuring approximately 23 × 16 mm in the left ovary, with well-defined margins, regular morphology, and satisfactory internal echogenicity, suggestive of an ovarian cyst. The patient's family declined surgical intervention. Subsequently, oral dienogest was prescribed, and follow-up examination demonstrated satisfactory recovery, after which the chest tube was removed and the patient was discharged.

Given that the pneumothorax episodes still occurred in relation to the menstrual cycle during this admission, a repeat gynecological consultation was obtained, and the therapeutic response to oral dienogest was considered suboptimal. The treatment was therefore switched to a goserelin acetate sustained-release implant. Chest CT performed during this admission revealed a large right-sided pneumothorax with approximately 80% or more lung compression, for which emergency tube thoracostomy was performed. Routine biochemical investigations revealed no significant abnormalities. A chest radiograph obtained on June 29, 2026, demonstrated post-thoracostomy changes on the right side, with a residual pulmonary compression margin devoid of lung markings, involving an estimated 40% to 50% of the lung parenchyma. The chest tube was visible in the right hemithorax. On July 2, 2026, air bubble leakage from the chest tube was still observed during coughing (Figure 3A). Given the clinical impression of a refractory air leak with poor healing potential, after detailed discussion with the patient's family, a single-port VATS procedure comprising right thoracic exploration, wedge resection of the lung, and pleural symphysis was performed on July 6, 2026. Intraoperative findings included a distinct air leak at the lateral segment of the right middle lobe, which was resected by wedge excision. Diaphragmatic fenestration (Figure 3B) and the leaking site at the lateral segment of the right middle lobe (Figure 3C) were also identified intraoperatively. Postoperative histopathological examination of the resected specimen (reported as bullae) revealed alveolar fusion and disruption, with focal aggregates of hemosiderin-laden macrophages in the subpleural interstitium. Immunohistochemical staining demonstrated positivity for ER, Pax-8, and CD10 (Figure 4), a staining profile consistent with endometriotic foci. In conjunction with the patient's clinical history and manifestations, a diagnosis of catamenial pneumothorax was confirmed. Postoperatively, goserelin acetate sustained-release implant therapy was continued (One injection every 28 days, one vial per dose), and up to the time of manuscript submission, no recurrence of pneumothorax had been documented. The diagnostic and therapeutic timeline for Case 2 is presented in Figure 5.

**Figure 3:**
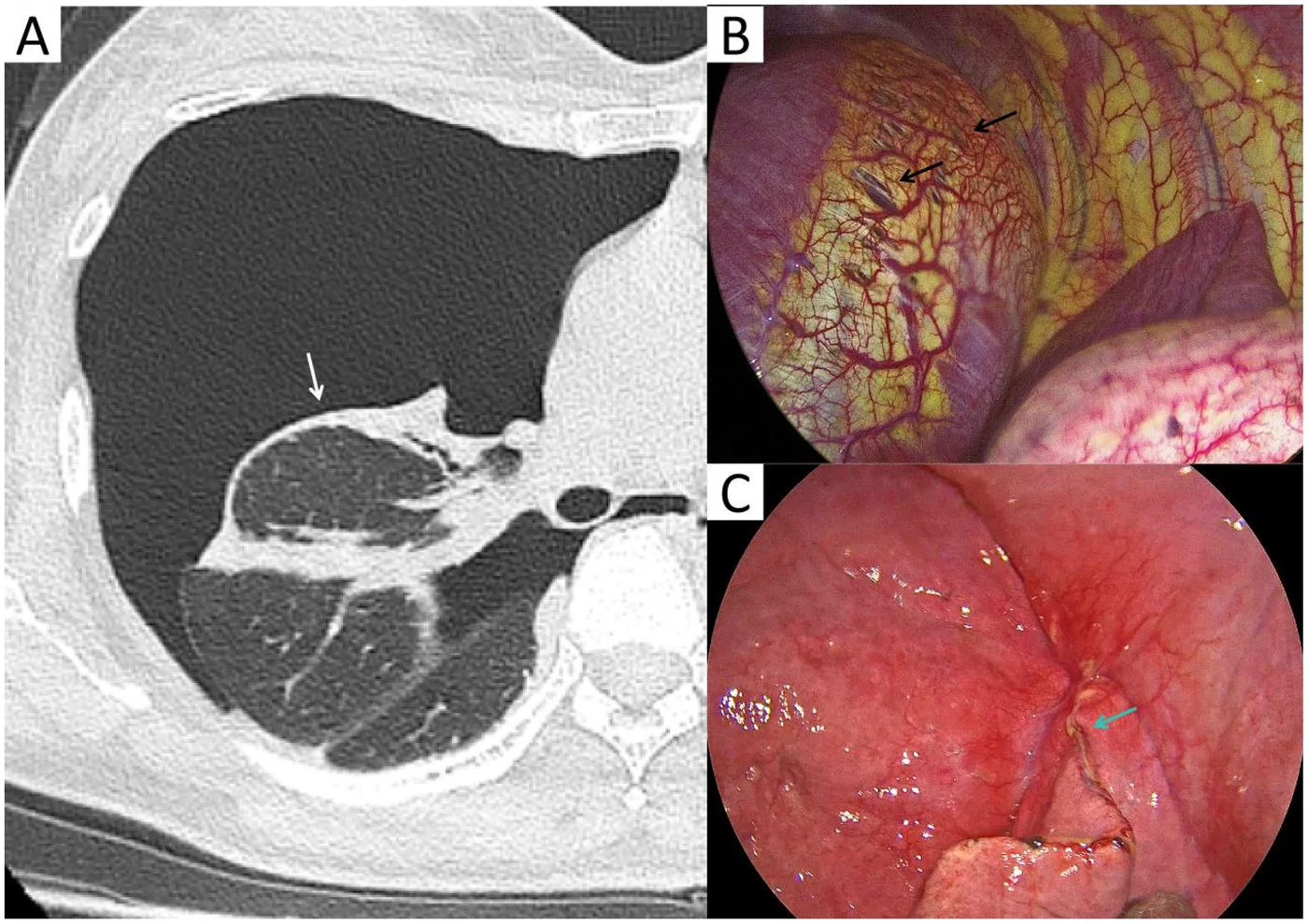
Imaging and intraoperative findings of case 2. **(A)** Chest CT demonstrates a large right-sided pneumothorax, with the white arrow indicating the margin of pulmonary compression. **(B)** Intraoperative exploration reveals multiple diaphragmatic fenestrations, as indicated by the black arrows. **(C)** The surgical field shows the site of air leakage identified during exploration, denoted by the blue arrow.

**Figure 4:**
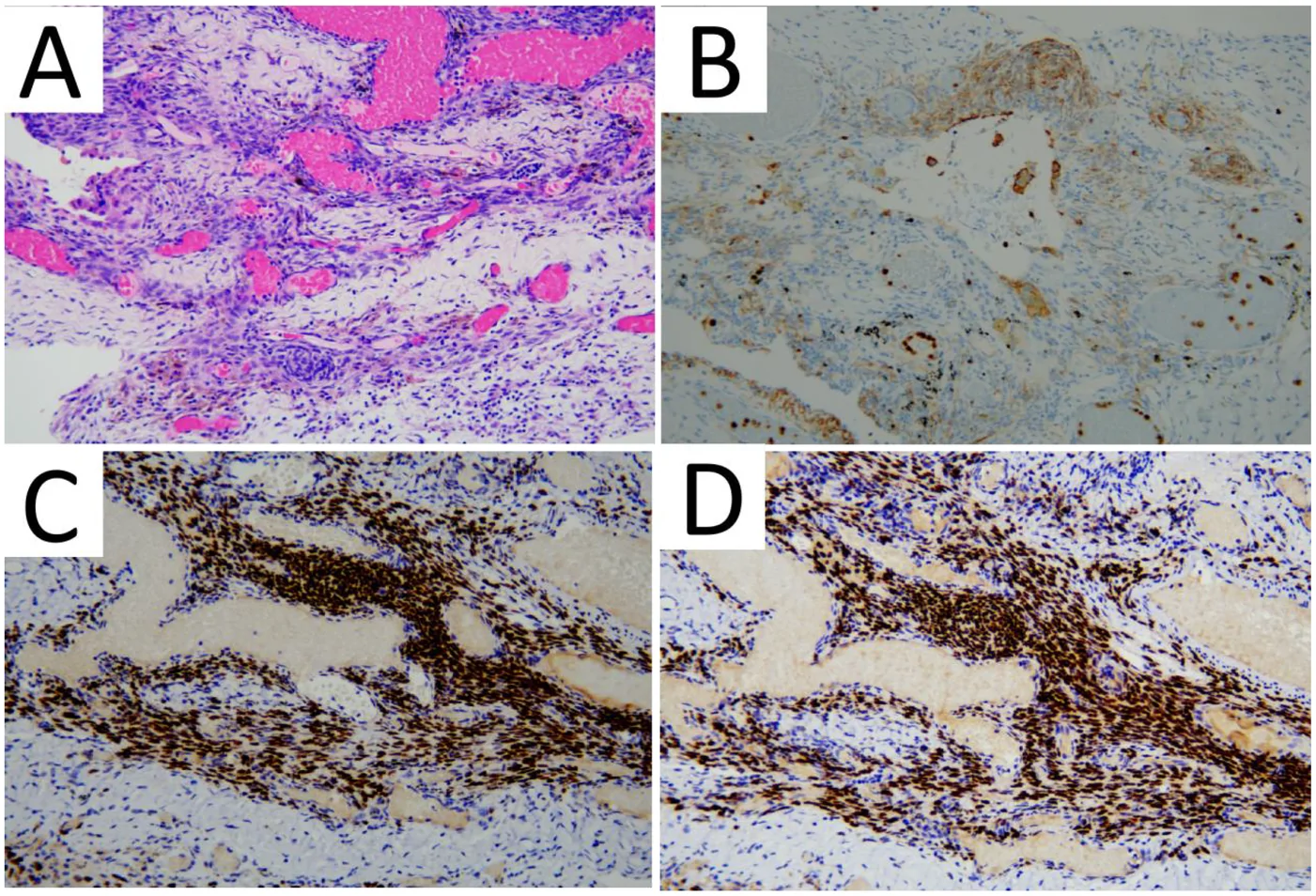
Histopathological and immunohistochemical findings of case 2. **(A)** Subpleural aggregation of spindle-shaped or oval-shaped cells with thin-walled capillaries and extravasated erythrocytes, accompanied by focal deposition of hemosiderin-laden macrophages (hematoxylin and eosin [HE] stain, ×200). **(B)** Immunohistochemistry (IHC) demonstrates positivity for CD10 in the spindle-shaped or oval-shaped cells (IHC, ×200). **(C)** IHC shows estrogen receptor (ER) positivity in the spindle-shaped or oval-shaped cells (IHC, ×200). **(D)** IHC reveals progesterone receptor (PR) positivity in the spindle-shaped or oval-shaped cells (IHC, ×200).

**Figure 5:**
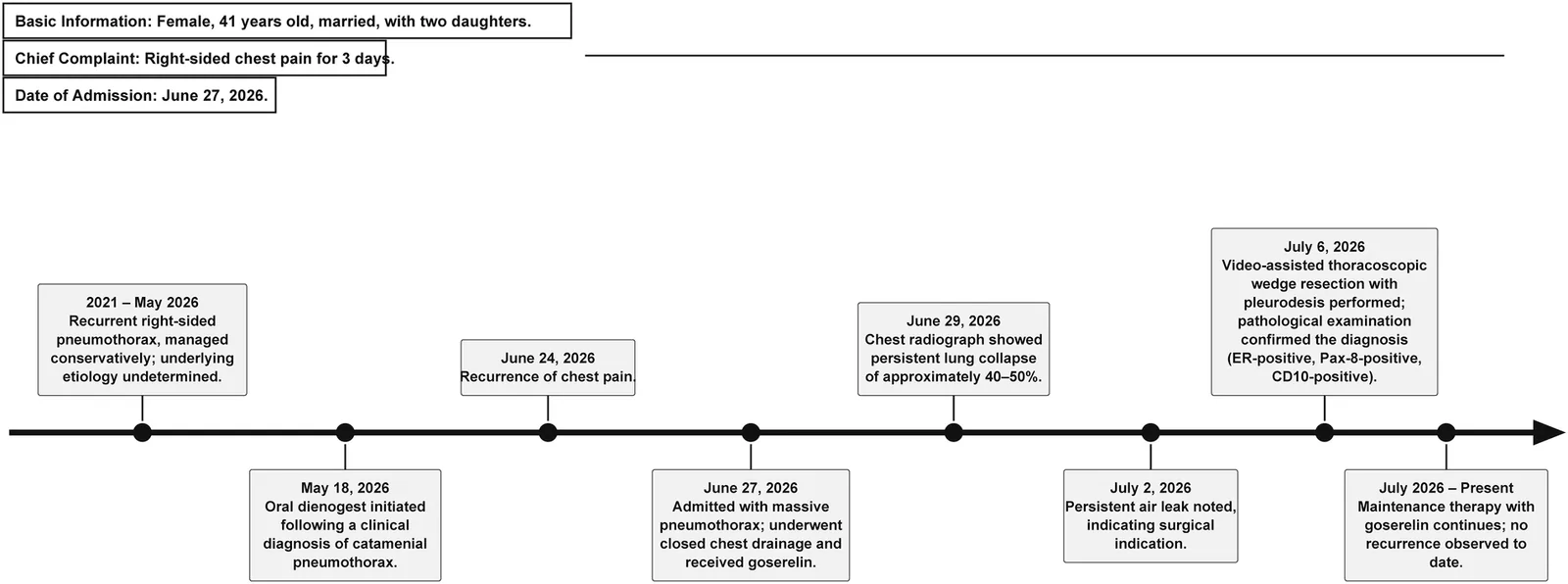
Timeline of the clinical course of case 2.

### 2.3 Comparison of the two cases

In Case 1, the diagnosis of catamenial pneumothorax was not considered at the initial presentation. The patient experienced recurrent pneumothorax postoperatively, with an increased volume of pneumothorax compared with the preoperative status, which posed a potential medicolegal concern. A definitive diagnostic direction was established only after multidisciplinary consultation involving the Department of Gynecology, leading to a clinical diagnosis of catamenial pneumothorax. Following empirical therapy, the clinical response was favorable; however, recurrence of pneumothorax was still observed upon resumption of menstruation after drug discontinuation. Drawing on the experience gained from Case 1, the possibility of catamenial pneumothorax was taken into account at the initial evaluation of Case 2. The diagnosis was ultimately confirmed by histopathological examination of the surgically resected specimen, which demonstrated endometriotic foci, and the findings were consistent with catamenial pneumothorax when correlated with the clinical presentation. A favorable outcome was achieved following combined surgical and endocrine therapy. A comparative summary of the two cases is presented in Table 1.

**Table 1:** Comparison of clinical characteristics between the two cases.

Feature	Case 1	Case 2
Age (years)	37	41
Time to diagnosis (years)	5	3
Clinical manifestations	Chest pain, chest tightness, dyspnea	Chest pain, chest tightness, dyspnea
Marital and obstetric history	Unmarried, nulliparous	Married, mother of two daughters
Past medical history	Postoperative status of thyroid carcinoma	Postoperative status of thyroid carcinoma
History of endometriosis	Present	Present, with a history of surgery for “ovarian cyst” six years prior
Surgical procedure	Single-port VATS wedge resection of the lung	Single-port VATS wedge resection of the lung with pleural symphysis
Diaphragmatic fenestration	Not explored	Present
Endocrine therapeutic agents	Leuprorelin acetate	Dienogest; goserelin acetate sustained-release implant
Postoperative immunohistochemical findings	ER (approximately 20% of stromal cells, weak-to-moderate intensity positive); PR (approximately 40% of stromal cells, moderate intensity positive)	ER (+), Pax-8 (+), CD10 (+)
Follow-up	Recurrence observed after drug discontinuation	No recurrence documented during ongoing maintenance therapy

## 3 Discussion

Pneumothorax is defined as the presence of air within the pleural cavity. It may result from rupture of pulmonary bullae or, more commonly, from traumatic injury, surgical procedures, positive-pressure ventilation, thoracic puncture, or catheterization of the cardiovascular system, which may lead to disruption of the lung, trachea, bronchus, or esophagus with subsequent air leakage into the pleural space. Alternatively, pneumothorax may occur when a chest wall wound penetrates the pleura, establishing a communication between the pleural cavity and the external environment, thereby allowing atmospheric air to enter. Endometriosis is a pathological condition characterized by the presence of functional endometrial tissue outside the uterine cavity, most frequently affecting women of reproductive age. Based on the anatomical sites of endometrial implantation, endometriosis can be classified into intrinsic and extrinsic types. Catamenial pneumothorax, also termed thoracic endometriosis-related pneumothorax, is a rare subtype of spontaneous pneumothorax and represents a form of extrinsic endometriosis.

The pathogenesis of catamenial pneumothorax remains incompletely understood, and several hypotheses have been proposed. One theory suggests that proteolysis of diaphragmatic endometriotic lesions, combined with chronic respiratory movement-induced tearing, may lead to the formation of diaphragmatic fenestrations, through which intra-abdominal air migrates into the thoracic cavity (1).

An alternative hypothesis posits that intrapleural implantation of endometrial tissue undergoes cyclic breakdown in response to menstrual hormonal fluctuations, resulting in local pleural disruption and bronchopleural fistula formation, thereby allowing intrapulmonary air to escape into the pleural space (11, 12). In the absence of identifiable diaphragmatic defects, it has also been speculated that increased prostaglandin concentrations during ovulation may induce vasoconstriction and ischemic injury to pulmonary tissue, culminating in alveolar rupture and pneumothorax (5, 13). However, current evidence remains inconclusive, and it is plausible that multiple mechanisms coexist, warranting further investigation.

In Case 2, a preoperative clinical diagnosis of catamenial pneumothorax prompted thorough intraoperative exploration, which revealed multifocal fenestrations at the diaphragmatic tendinous portion—a location consistent with that most commonly reported in the literature (1). Additionally, thickened adhesions and prominent vascular structures were observed at the pulmonary apex, suggestive of pleural adhesion formation secondary to recurrent pneumothorax. During intraoperative saline irrigation with lung inflation, a distinct air leak was identified at the lateral segment of the right middle lobe; wedge resection of this pulmonary tissue subsequently yielded histopathological confirmation of endometriotic tissue. The clinical manifestations—including chest pain, chest tightness, and dyspnea—were consistent with those of typical pneumothorax. The following discussion focuses on the clinical characteristics and diagnostic-therapeutic strategies for catamenial pneumothorax.

The onset of catamenial pneumothorax is frequently and closely associated with the menstrual cycle, which constitutes the most critical distinguishing feature from other types of pneumothorax. Episodes typically occur within 24 to 72 hours before or after the onset of menstruation, and the recurrence rate is notably high (1, 5–7). The reported prevalence accounts for approximately 0.9% of all spontaneous pneumothoraces in women, although this proportion may rise to as high as 35% in young female populations (14). From a pathophysiological perspective, this temporal pattern is intimately linked to the dramatic fluctuations in hormone levels during the menstrual cycle. In the premenstrual phase, the abrupt decline in estrogen and progesterone levels induces withdrawal necrosis and shedding of ectopically implanted endometrial tissue within the thoracic cavity. When such lesions involve the visceral pleura or the diaphragmatic surface, pleural disruption and air leakage may ensue. Catamenial pneumothorax is predominantly unilateral, with over 80% of cases affecting the right side (15). The pathophysiological basis for this right-sided predominance is thought to relate to the clockwise circulation of peritoneal fluid. Under the influence of diaphragmatic motion and intestinal peristalsis, free intra-abdominal fluid—including retrograde menstrual debris—tends to flow upward along the right paracolic gutter toward the right subphrenic space, thereby preferentially exposing the right hemidiaphragm to endometrial fragments and increasing the likelihood of endometriotic implantation on the right side. Furthermore, the bare area of the liver is located immediately beneath the right hemidiaphragm; in the presence of congenital or acquired diaphragmatic defects, intra-abdominal air and endometrial tissue may thus gain direct access to the right pleural cavity.

The two patients in the present study were aged 41 and 37 years, respectively, both of whom were women of reproductive age, and the pneumothorax occurred on the right side in both cases, which is consistent with previous reports in the literature. Both patients had a history of recurrent right-sided pneumothorax, with diagnostic delays of five and three years, respectively, which substantially compromised their quality of life. In Case 1, the diagnosis was not established until after multiple postoperative recurrences had occurred; endocrine therapy targeting endometriosis yielded a certain degree of clinical response, although recurrence of pneumothorax was again observed upon drug discontinuation. We therefore recommend uninterrupted hormone therapy following surgical intervention (9). However, several clinically relevant factors should be taken into consideration. Some patients remain unmarried and nulliparous, and the current trend of delayed childbearing further complicates decision-making. Moreover, women with endometriosis have a lower natural pregnancy rate and a higher incidence of infertility compared with the general population. Although the “medical menopause” state induced by GnRH agonists can effectively suppress ectopic endometrial activity, it simultaneously precludes the possibility of natural conception, thereby posing practical challenges in clinical implementation. Assisted reproductive technology (16) may also be considered as one of the available options.

The natural history of catamenial pneumothorax is characterized by a high recurrence rate. When palliative measures—such as simple thoracentesis or tube thoracostomy—are employed without addressing the underlying etiology, the recurrence rate approaches 100%. Even when VATS with bullectomy and pleural symphysis is performed, the postoperative recurrence rate may remain as high as 30% to 50% if intraoperative exploration fails to examine the diaphragm and manage endometriotic lesions or diaphragmatic defects (17). Therefore, when catamenial pneumothorax is clinically suspected, comprehensive intraoperative exploration of the thoracic cavity is recommended. In cases where no obvious air leak is identified in the pulmonary parenchyma, careful inspection of the diaphragm should be undertaken. If diaphragmatic defects or fenestrations are detected—particularly in patients presenting with recurrent postoperative pneumothorax—diaphragmatic repair is advised. Small defects may be managed by direct suture or plication using non-absorbable sutures; larger defects or areas of diaphragmatic attenuation may be repaired with synthetic or biological mesh for tension-free reconstruction (5, 18), and when necessary, poly-lactic acid mesh may be applied to cover the diaphragmatic surface (19). However, ectopic endometrial tissue on the pleural surface remains difficult to identify, and residual pleural endometrial deposits may confer an additional risk of pneumothorax recurrence. Accordingly, postoperative endocrine therapy is recommended in conjunction with surgical intervention to reduce the likelihood of recurrence (5, 15, 17, 20). Interestingly, both cases had a concomitant history of thyroid cancer and were on long-term oral thyroxine therapy; however, no clear association between these conditions and endometriosis has been reported in the literature.

Endocrine therapy exerts its effect by suppressing ovarian function and attenuating the hormonal fluctuations associated with the menstrual cycle, thereby inducing atrophy of ectopic endometrial tissue and consequently reducing the risk of pneumothorax recurrence. Commonly used therapeutic agents include the following:

GnRH agonists: By inducing pituitary desensitization, these agents suppress ovarian estrogen secretion and produce a reversible state of “medical menopause,” which is currently the most effective and most commonly employed endocrine treatment modality, leading to marked atrophy of ectopic endometrial tissue. The main adverse effects are hypoestrogenic symptoms (e.g., hot flushes and risk of osteoporosis), which may be partially alleviated by add-back therapy with low-dose estrogen-progestin combinations.

Oral contraceptives: Continuous administration of combined oral contraceptives (avoiding withdrawal bleeding) suppresses ovulation and stabilizes the endometrium, and may be used in mild cases or as maintenance therapy following surgery.

Progestogens: High-potency progestogens (e.g., dienogest (21)) induce decidualization and atrophy of ectopic endometrial tissue, and are suitable for patients who have indications for endocrine therapy but are not candidates for GnRH agonists.

Postoperatively, combined endocrine therapy is recommended for a minimum of six months to consolidate the surgical outcome and reduce the risk of early recurrence (20). The specific regimen should be tailored to the individual patient, taking into account the severity of symptoms, extent of lesions, reproductive wishes, drug tolerance, and economic considerations, and should be formulated jointly by a gynecological endocrinologist and a thoracic surgeon.

Although catamenial pneumothorax is a relatively rare entity, a thorough understanding of its clinical characteristics and the formulation of rational and efficient diagnostic and therapeutic strategies remain of considerable clinical importance. The combination of female reproductive age, recurrent right-sided pneumothorax, and a temporal association with the menstrual cycle may serve as a “triad” of warning signs for catamenial pneumothorax. In the diagnostic workup, a multidisciplinary approach integrating input from both thoracic surgeons and gynecologists is strongly recommended. The diagnostic process should incorporate etiological background, clinical presentation, pattern of recurrence, and imaging findings; when clinically indicated, a detailed gynecological history and pelvic ultrasonography should also be obtained. If surgical intervention is undertaken, meticulous intraoperative exploration of the chest wall, lung parenchyma, and diaphragmatic surface should be performed to facilitate early detection of endometriotic lesions. Postoperatively, a multimodal management strategy should be individualized according to the patient's specific circumstances. Resected specimens should be referred to the Department of Pathology for endometriosis-directed histopathological evaluation. Multidisciplinary collaboration between thoracic surgery and obstetrics/gynecology, combined with endocrine therapy, is recommended to reduce the recurrence rate. The two cases reported herein both experienced recurrent episodes and multiple hospital visits, underscoring the critical importance of enhancing clinical awareness and recognition of this condition. In women of reproductive age presenting with recurrent right-sided pneumothorax, a detailed menstrual history should be routinely elicited as part of the standard medical interview to avoid misdiagnosis as simple pneumothorax and to prevent delays in targeted therapy, thereby improving treatment outcomes. Furthermore, given the potential for long-term recurrence, regular and structured follow-up is advised for all patients with a confirmed diagnosis.

This is a retrospective case series with inherent limitations. For Case 1, only ER/PR immunostaining was positive; further confirmatory testing for endometriosis was not performed. Therefore, the diagnosis of thoracic endometriosis was established by exclusion of other diseases and positive response to hormonal treatment, rather than definitive pathological proof. Moreover, intraoperative diaphragmatic exploration and pleurodesis were not performed in this patient. We have stated that clinicians should adopt a more comprehensive evaluation strategy when encountering similar cases in future practice.

## 4 Conclusion

In women of reproductive age presenting with recurrent right-sided pneumothorax accompanied by chest pain, chest tightness, and dyspnea, particularly when the onset of symptoms is temporally related to the menstrual cycle, a high index of suspicion for catamenial pneumothorax should be maintained. Clinicians are encouraged to reinforce the diagnostic principle that a detailed menstrual history must be routinely obtained in women of reproductive age with recurrent pneumothorax, in order to reduce missed diagnoses and misdiagnoses and improve patient outcomes. Conservative hormone therapy may be considered as the first-line treatment. In cases of persistent air leakage requiring surgical intervention, thorough intraoperative exploration of the diaphragm and pleura should be performed, and resected specimens should be submitted for immunohistochemical evaluation to aid in the diagnosis of endometriosis. When indicated, diaphragmatic repair and pleural symphysis should be undertaken. Postoperatively, adjunctive endocrine therapy is recommended to reduce the recurrence rate. Close collaboration with a gynecologist is advised throughout the entire diagnostic and therapeutic course.

## Data Availability

The original contributions presented in the study are included in the article/Supplementary Material, further inquiries can be directed to the corresponding author.
